# Extracellular vesicles as advanced therapeutics for the resolution of organ fibrosis: Current progress and future perspectives

**DOI:** 10.3389/fimmu.2022.1042983

**Published:** 2022-10-20

**Authors:** Ke Lv, Yizhuo Wang, Peng Lou, Shuyun Liu, Pingya Zhou, Li Yang, Yanrong Lu, Jingqiu Cheng, Jingping Liu

**Affiliations:** ^1^ National Health Commission (NHC) Key Laboratory of Transplant Engineering and Immunology, Regenerative Medicine Research Center, Frontiers Science Center for Disease-related Molecular Network, West China Hospital, Sichuan University, Chengdu, China; ^2^ Department of Gastroenterology and Hepatology, Sichuan University-University of Oxford Huaxi Joint Centre for Gastrointestinal Cancer, West China Hospital, Sichuan University, Chengdu, China

**Keywords:** fibrosis, extracellular vesicles, exosomes, nanomedicine, biomaterials

## Abstract

Organ fibrosis is a serious health challenge worldwide, and its global incidence and medical burden are increasing dramatically each year. Fibrosis can occur in nearly all major organs and ultimately lead to organ dysfunction. However, current clinical treatments cannot slow or reverse the progression of fibrosis to end-stage organ failure, and thus advanced anti-fibrotic therapeutics are urgently needed. As a type of naturally derived nanovesicle, native extracellular vesicles (EVs) from multiple cell types (*e.g.*, stem cells, immune cells, and tissue cells) have been shown to alleviate organ fibrosis in many preclinical models through multiple effective mechanisms, such as anti-inflammation, pro-angiogenesis, inactivation of myofibroblasts, and fibrinolysis of ECM components. Moreover, the therapeutic potency of native EVs can be further enhanced by multiple engineering strategies, such as genetic modifications, preconditionings, therapeutic reagent-loadings, and combination with functional biomaterials. In this review, we briefly introduce the pathology and current clinical treatments of organ fibrosis, discuss EV biology and production strategies, and particularly focus on important studies using native or engineered EVs as interventions to attenuate tissue fibrosis. This review provides insights into the development and translation of EV-based nanotherapies into clinical applications in the future.

## 1 Introduction

**Figure 1:**
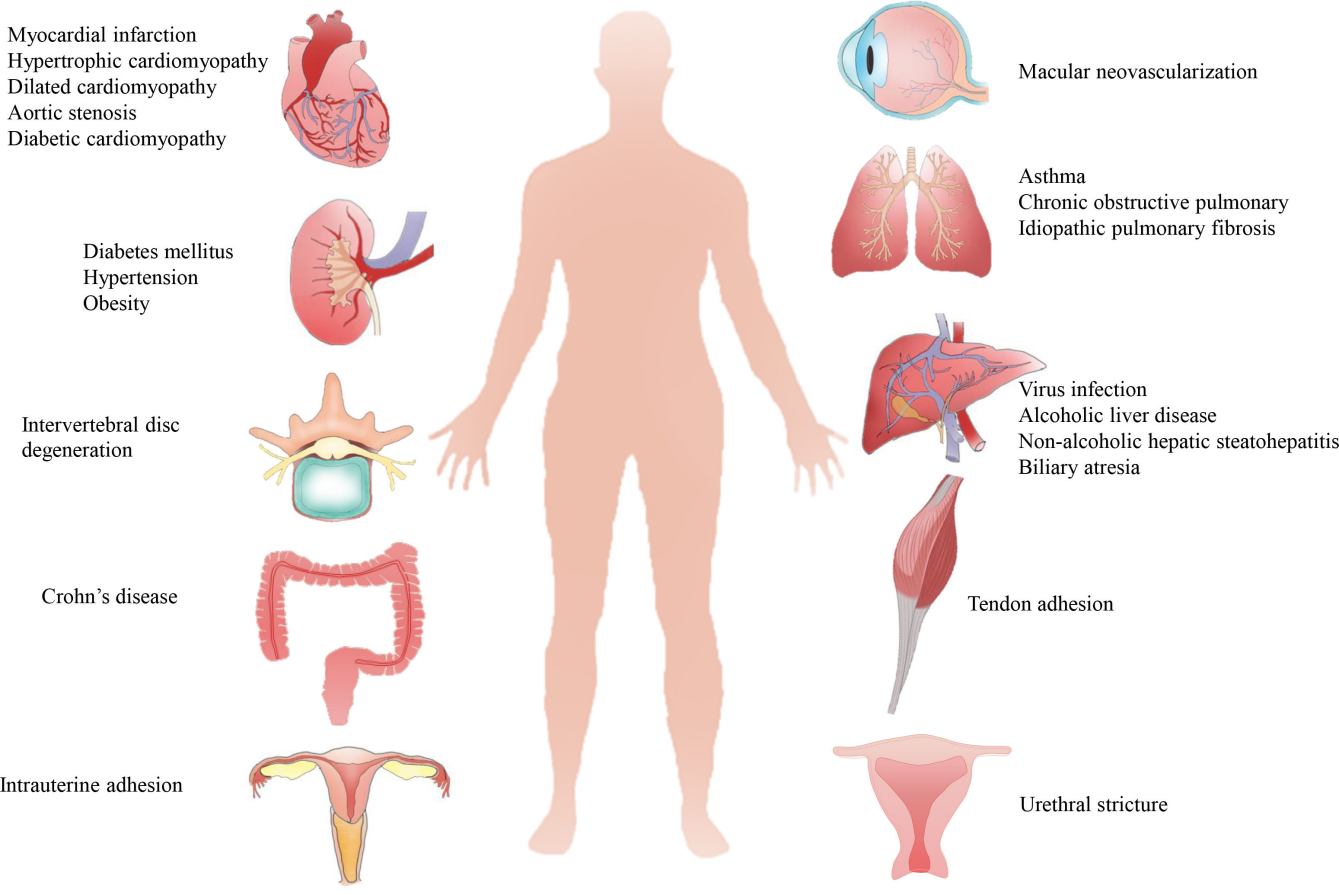
Pathological causes of organ fibrosis. Fibrosis can occur in most organs or tissues, such as the heart, liver, lung, kidney, tendon, intrauterine and intervertebral disc, due to complicated pathological causes.

Organ fibrosis is a serious and unsolved health problem worldwide. The main pathological feature of fibrosis is the abnormal formation and deposition of excessive extracellular matrix (ECM), which eventually results in disrupted architectural remodeling and progressive organ dysfunction. Fibrosis can occur in nearly all major organs (**Figure 1**), such as liver, heart, lung, and kidney, and it is attributed to ~45% of all deaths in the world (1). For example, more than 800 million patients were affected by chronic liver disease (liver fibrosis); about 1.5% of global deaths were caused by chronic kidney disease (CKD, renal fibrosis) in 2012 (2, 3). The mechanism of fibrosis is extremely complicated, and many pathological factors, such as infections, immune reactions, chemical insults, oxidative stress, and hazardous agents, have been proved to be involved with fibrosis (4). In recent decades, many single factor-targeted treatments have been developed and have shown certain beneficial effects in preclinical studies, but most of them fail to achieve clinical approval. Thus, novel and advanced antifibrotic therapeutics are desired in the clinic (5).

In recent years, extracellular vesicle (EV)-based therapies have emerged as a potent strategy for resolving organ fibrosis. EVs are nanoscale bilayer vesicles secreted by live cells that exert similar functions as parental cells by delivering various types of cargoes, such as lipids, proteins, nucleic acids, and metabolites (6). As a type of naturally derived nanomaterial, EVs have multiple advantages, such as abundant cell sources, intrinsic bioactive properties, low immunogenicity, rare toxicity, flexibility to modify, and ability to cross biological barriers compared to synthetic materials (7). In many preclinical studies, EV-based nanomedicines have been shown to prevent multiple types of organ fibrosis through complicated mechanisms, such as promoting the recovery of damaged tissues, resolution of inflammation, inactivation of myofibroblasts, and fibrinolysis of excess ECM components (5). Although the results are encouraging, some important problems, such as proper cell sources, EV engineering strategies, detailed mechanisms, and possible limitations, remain elusive and need to be comprehensively reviewed. This will be helpful for the improvement and clinical translation of EV-based antifibrotic therapies.

In this review, we briefly introduce the key pathology of organ fibrosis as well as EV biology and production and emphasize the important studies that are highly relevant to using native or engineered EVs for decreasing organ fibrosis. We also discuss the possible limitations in this field and provide insights into developing advanced EV therapeutics for the treatment of fibrotic diseases.

## 2 Pathology and current therapies of fibrosis

**Figure 2:**
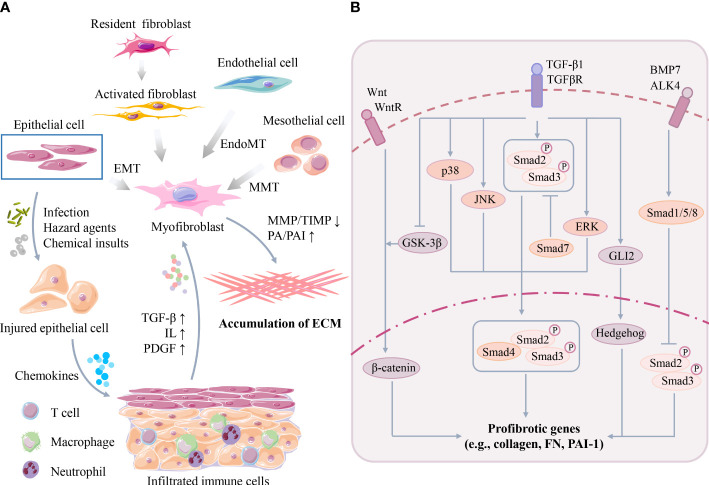
**(A)** Key cellular events of organ fibrosis. Organ damage can trigger infiltration of immune cells, followed by secretion of excessive cytokines/chemokines to activate myofibroblasts. Myofibroblasts are originated from many cell types, such as epithelial cells, mesothelial cells and endothelial cells, through EMT, MMT or EndoMT. Activated myofibroblasts promote organ ECM synthesis/deposition. **(B)** Key signaling pathway of organ fibrosis. TGF-β participates in the molecular mechanism of fibrosis in Smad-dependent and Smad-independent manner such as MAPK families. Moreover, TGF-β also interacts with other profibrotic pathways, such as the Wnt/β-catenin, Hedgehog and BMP-7 pathways. (TGF-β, transforming growth factor-β; ILs, interleukins; PDGF, platelet-derived growth factor; EMT, epithelial to mesenchymal transition; MMT, mesothelial to mesenchymal transition; EndoMT, endothelial to mesenchymal transition; MMP, matrix metalloproteinase; TIMPs, tissue inhibitors of matrix metalloproteinases; PA, plasminogen activator; PAI, plasminogen activator-inhibitor 1; MAPK, mitogen-activated protein kinase; JNK, JUN amino-terminal kinase; ERK, extracellular signal-regulated kinase; GSK-3β, glycogen synthase kinase-3β; FN, fibronectin).

Fibrosis, characterized by the activation of myofibroblasts, excessive deposition of ECM, and inhibition of ECM degradation, is a common adverse outcome of many etiological conditions after acute or chronic organ injury (8, 9). A variety of cell types and signaling pathways synergistically regulate the occurrence and progression of organ fibrosis (**Figure 2**). In this section, we briefly review the critical cellular events, signaling pathways and current clinical strategies for fibrotic disease treatment.

### 2.1 Key cellular events of fibrosis

In the early phases after organ injury, persistent stress may induce parenchymal cell death (*e.g.*, necrosis, pyroptosis, and ferroptosis) and trigger abnormal activation and infiltration of multiple types of immune cells (*e.g.*, macrophages) and inflammation (10). Subsequently, cytokines and chemokines secreted by these infiltrated immune cells, such as transforming growth factor-β (TGF-β), interleukins (ILs), and platelet-derived growth factor (PDGF), further amplify the severity of inflammation and organ damage (11). The activation of myofibroblasts is the central event mediating ECM synthesis and deposition in the later phases after organ injury. Activated myofibroblasts can be identified by several marker proteins, such as α-smooth muscle actin (α-SMA) and PDGFβR (12). However, the origins of myofibroblasts remain incompletely understood, but multiple cell sources have been reported (**Figure 2A**), such as resident fibroblasts, mesothelial cells, circulating fibroblasts, epithelial cells, endothelial cells, pericytes, vascular smooth muscle cells, and other specialized tissue cells (*e.g.*, hepatic stellate cells). Additionally, many possible mechanisms, such as cell proliferation, epithelial to mesenchymal transition (EMT), mesothelial to mesenchymal transition (MMT) and endothelial to mesenchymal transition (EndoMT), are involved in this process (5, 13).

The imbalance between ECM deposition and ECM degradation is another vital event during fibrosis. Briefly, the degradation of ECM proteins (*e.g.*, collagens) is mainly controlled by matrix metalloproteinase (MMP)-mediated proteolysis but is antagonized by multiple tissue inhibitors of matrix metalloproteinases (TIMPs), which are the endogenous inhibitors of MMPs (14, 15). MMPs can be classified into different subtypes, such as interstitial collagenases, gelatinases, metalloelastases and membrane-type MMPs, according to their enzyme-substrate specificity and subcellular locations (16). For example, interstitial collagenases (*e.g.*, MMP-1, MMP-13, and MMP-18) can proteolyze type I, II, III interstitial collagens, while gelatinases (*e.g.*, MMP-2 and MMP-9) can cleave the denatured collagens and basement membrane ECM (16, 17). Conversely, TIMPs can blunt ECM proteolysis by inhibiting the activity of MMPs, and the upregulation of TIMPs is commonly associated with an accumulation of ECM (18). In addition, the dysregulation of serine proteases and their inhibitors, such as urokinase plasminogen activator (uPA) and plasminogen activator-inhibitor 1 (PAI-1), is also associated with abnormal ECM remodeling and fibrosis (14).

### 2.2 Key signaling pathways of fibrosis

Although many possible pathways have been revealed in the pathogenesis of organ fibrosis in recent decades, TGF-β has been recognized as a master regulator of myofibroblast activation and fibrotic processes (**Figure 2B**). TGF-β has three isoforms (TGFβ1-3) and can stimulate the biosynthesis and accumulation of ECM components, such as collagen I and fibronectin (FN), in epithelial cells and mesenchymal cells (19, 20). TGF-β1 can be secreted by many types of cells, such as macrophages, lymphocytes, epithelial cells, fibroblasts, pericytes, endothelial cells and platelets, at injured tissue sites (19). Once activated after secretion, TGF-β1 binds to its receptor (TGF-βR) and activates downstream profibrotic pathways in a Smad-dependent (classical) or Smad-independent (nonclassical) manner (21). In the Smad-dependent pathway, TGF-β1 induces phosphorylated Smad2/3 proteins, and then Smad4 binds to the Smad2/3 complex and translocates the complex into the nucleus to induce the transcription of many essential profibrotic genes, such as collagens, FN and PAI-1 (9). Smad7 is a negative regulator of this process, which can compete with Smad2/3 for binding to activated TGF-βR and thus block TGF-β/Smad signaling (22). Moreover, TGF-β1 can also interact with other Smad-independent pathways, such as the mitogen-activated protein kinase (MAPK) family p38 MAPK, JUN amino-terminal kinase (JNK) and extracellular signal-regulated kinase (ERK) pathways. MAPKs further phosphorylate the linker region of Smad2/3 and thus modulate Smad3 transcriptional activity (9).

Furthermore, TGF-β has potential crosstalks with other profibrotic pathways, such as the Wnt/β-catenin, Jagged1/Notch, Hedgehog, and bone morphogenic protein-7 (BMP-7) pathways (23). For example, TGF-β activates Wnt signaling by inhibiting glycogen synthase kinase-3β (GSK-3β), thereby disrupting the stabilization of β-catenin or suppressing Wnt inhibitors and enhancing the transcription of β-catenin-targeted profibrotic genes (*e.g.*, FN, PAI-1, Snail and MMP-7) in injured kidneys (24, 25). TGF-β can also induce the transcription of GLI2 (an activator of Hedgehog signaling), which subsequently upregulates Hedgehog-targeted profibrotic gene (*e.g.*, α-SMA) expression (26–28). In contrast, BMP-7 activates Smad1/5/8, which can block the nuclear translocation of phosphorylated receptor-Smad2/3 and thus inhibit TGF-β signaling (29). Due to the complicated mechanisms of organ fibrosis, it can be speculated that single pathway-targeted antifibrotic strategies may not be efficient.

### 2.3 Current antifibrotic strategies

Since fibrosis is a long-term outcome of persistent organ damage, it can be assumed that the resolution of fibrosis may be achieved when the pathological causes of injuries are eliminated. Based on previous studies of fibrogenesis, several therapeutic strategies (**Table 1**) are proposed (30), such as immunoregulation, degradation of ECM and elimination of myofibroblasts. Currently, some potential targets or medicines for fibrosis in different organs have been reported, and some of them have progressed to clinical trials or clinical phases (**Table 1**). For example, pirfenidone and nintedanib (NIN), two compounds with pleiotropic mechanisms of action, are approved for the management of idiopathic pulmonary fibrosis (IPF) due to their effects on slowing lung functional decline. Pirfenidone may inhibit TGF-β, inflammatory cytokines (*e.g.*, tumor necrosis factor-α, TNF-α) or oxidative stress, and NIN may inhibit tyrosine kinase receptors such as PDGFR, vascular endothelial growth factor receptor (VEGFR) and fibroblast growth factor receptor (FGFR) (31). However, the two drugs cannot prevent or reverse the existing organ fibrosis according to physiological, histological, and radiological examination results (31). Thus, more advanced antifibrotic treatments are urgently required in the clinic.

**Table 1:** Current anti-fibrotic strategies and drugs.

Strategies	Drugs	Classes	Targets/mechanisms	Target diseases	Phase
Myofibroblasts and the TGF-β pathway	Pirfenidone	stress-activated kinases inhibitor	TGF-β, PDGF, SDF-1α	IPF, pulmonary fibrosis, hepatic fibrosis, renal fibrosis, systemic sclerosis, keloid	Clinic
	Nintedanib (BIBF 1120)	tyrosine kinase inhibitor	TGF-β, PDGF, VEGF, FGF	IPF, systemic sclerosis	Clinic
	Imatinib mesylate (Glivec)	tyrosine kinase inhibitor	PDGF	IPF, pulmonary fibrosis, hepatic fibrosis, nephrogenic systemic fibrosis, systemic sclerosis	1/2/3
	Pamrevlumab (FG-3019)	human recombinant monoclonal antibody	CTGF	IPF	2/3
	Macitentan	endothelin receptor antagonist	endothelin-1	IPF	2
	BMS-986263	etinoid-conjugated lipid nanoparticle containing HSP47 siRNA	HSP 47	hepatic fibrosis, cirrhosis, NASH	1/2
ECM	STX-100	specific monoclonal antibody	αvβ6 integrin	IPF, chronic allograft dysfunction	2
Immunomodulators	TD139	thio-digalactoside inhibitor	galectin-3	IPF	1/2
	Belapectin (GR-MD-02)	Galectin inhibitor	galectin-3	NASH, cirrhosis	2
	PRM-151	monocyte development inhibitor	Pentraxin 2	IPF, primary myelofibrosis, post-essential thrombocythemia myelofibrosis	1/2/3
	Spironolactone	aldosterone antagonists	Anti-inflammatory	myocardial fibrosis, endomyocardial fibrosis, hepatic fibrosis, cirrhosis, renal fibrosis, ESRD	2/3/4
	Lenabasum	cannabinoid type 2 receptor (CB2) agonist	Anti-inflammatory	cystic fibrosis	2
	Digitoxin	cardiac glycosides	IL-18/NF-κB	cystic fibrosis	2
	Pentoxifylline	anticytokine	TNF-α	head and neck fibrosis, radiation injuries, cirrhosis, NASH, CKD	1/2/3/4
Antioxidants	N-acetylcysteine (NAC)	antioxidant GSH prodrug	Oxidative stress	IPF, pulmonary fibrosis, cystic fibrosis, non-cystic fibrosis, cirrhosis, ESRD	1/2/3/4
Others	Aramchol	inhibitors of *de novo* lipogenesis	Hepatic SCD1	hepatic fibrosis, NASH	2/3
	Emricasan (IDN-6556)	caspase inhibitor	Apoptosis	hepatic fibrosis, cirrhosis, NASH	2

TGF-β, transforming growth factor-β; PDGF, platelet-derived growth factor; SDF-1α, stromal cell derived factor-1α; IPF, idiopathic pulmonary fibrosis; VEGF, vascular endothelial growth factor; FGF, fibroblast growth factor; CTGF, connective tissue growth factor; HSP47, heat shock protein 47; NASH, nonalcoholic steatohepatitis; ESRD, end stage renal disease; TNF-α, tumor necrosis factor-α; CKD, chronic kidney disease; SCD1, Stearoyl-CoA desaturase 1.

## 3 Biological properties of EVs

EVs are a group of nanoscale bilayer vesicles and are widely distributed in the cultured medium of almost all cell types and biofluids, such as blood, urine, saliva, and breast milk (32). Numerous evidence indicates that EVs are key mediators of cell-to-cell or organ-to-organ communication since they can deliver multiple types of bioactive cargoes to regulate the signaling of the recipient cells under physiological or pathophysiological conditions (33). In this section, we briefly introduce the biological properties of EVs, such as biogenesis, uptake, production, and stability.

### 3.1 EV biogenesis

**Figure 3:**
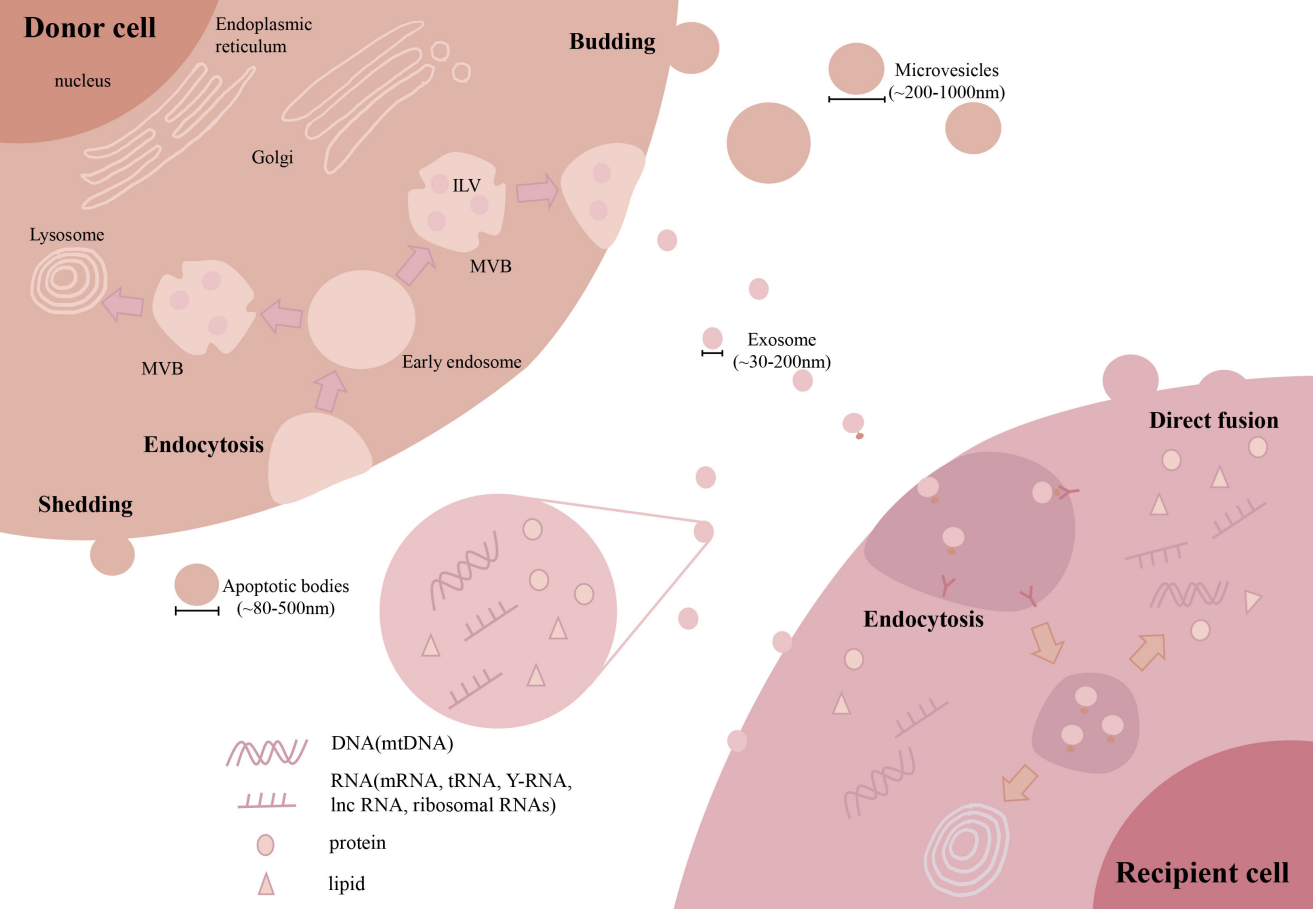
Biogenesis and uptake of EVs. During exosome biogenesis, the plasma membrane is endocytosed to form early endosomes and then matures into MVBs. The delimiting membrane of MVBs invaginates from the ILVs. Finally, some MVBs bind to the cell membrane to release ILVs. MVs are mainly originated by outward budding at the cell plasma membrane. For cellular uptake, EVs can be taken up by recipient cells *via* direct membrane fusion or endocytosis by interacting with receptors on the surface. (Extracellular vesicles: EVs, microvesicles: MVs, multivesicular body: MVB, intraluminal vesicles: ILV, microRNA: miRNAs, long noncoding RNA: lncRNA, mitochondrial DNA: mtDNA).

The current classification of EVs is mainly based on their cellular origin and/or biological function. According to their sizes or biogenesis routes, EVs can be divided into many subtypes, such as exosomes (~30-200 nm), microvesicles (MVs, ~200-1000 nm) and apoptotic bodies (~800-5000 nm) (34, 35). Among these EV subtypes, exosomes and MVs are frequently reported in most of the previous studies related to fibrosis. Thus, we will focus on these two types of EVs in the following sections (**Figure 3**). Briefly, the first step of exosome biogenesis is that the plasma membrane is endocytosed to form early endosomes, which then mature into late endosomes, also known as multivesicular bodies (MVBs). The delimiting membrane of MVBs can be invaginated to form intraluminal vesicles (ILVs), followed by the endosomal sorting complex required for transport (ESCRT)-dependent or ESCRT-independent (tetraspanins related) steps. In the ESCRT-dependent process, ESCRT-0 binds to the ubiquitination sites of early endosomes, ESCRT-I and ESCRT-II induce the formation of MVBs, and ESCRT-III promotes intraluminal budding of endosomal vesicles and scissors ILVs into the MVB lumen. The last step of exosome formation is the binding of some MVBs to the cell membrane to release ILVs, whereas other ILVs binds to lysosomes and are degraded (36). Unlike exosomes, MVs are mainly originated by outward budding at the cell plasma membrane, which is regulated by several rearrangements within the plasma membrane, such as altered lipid/protein components and Ca^2+^ levels. Although exosomes and MVs may have different biogenesis routes, they still share some common pathways, such as ESCRT and the conversion of ceramide from sphingomyelinas (37). However, the detailed mechanisms of other EV subpopulations remain elusive and need to be explored in future studies.

### 3.2 EV composition and uptake

EVs can participate in intracellular communication by delivering multiple types of bioactive contents into recipient cells (**Figure 3**). EVs are enriched in lipid contents, such as cholesterol, sphingomyelin, and hexosylceramides (38), which contribute to the *in vivo* stability of EVs. Due to their biogenesis routes, EVs carry many proteins originated from the cell plasma membrane, cytoskeleton and cytoplasm, as well as other proteins involved in EV sorting and secretion, such as tetraspanins and proteins from the ESCRT-dependent pathway (39). In addition, EVs also contain large amounts of RNAs and DNAs, among which microRNAs (miRNAs) are the most abundantly studied to explain the effective mechanisms of EVs. Other types of RNAs, such as mRNAs, long noncoding RNAs (lncRNAs), tRNAs, Y-RNAs and ribosomal RNAs, have also been observed in EVs (40, 41). Interestingly, we and others had found the appearance of mitochondrial DNA (mtDNA), electron transport chain proteins and even fragmented mitochondria in EVs (42).

As a result, the composition of proteins and lipids on the surface of EVs may influence the efficiency of cell uptake (**Figure 3**). In brief, EVs can be taken up by target cells *via* direct membrane fusion or endocytosis, which has been well reviewed (43). Direct fusion relies on the lipid composition of EVs and target cells, and the fusion efficacy of EVs to the plasma membrane may be enhanced by acidic pH in the extracellular environment (44). In the case of endocytosis, EVs are docked by interacting with proteins, glycoproteins or lipids exposed on the cell membrane and then internalized by recipient cells (45). Overall, the surface signature of EVs can influence the pattern of EV uptake in target cells.

### 3.3 EV isolation and characterization

EVs can be isolated from various types of samples (*e.g.*, conditioned medium (CM) and body fluids) using different methods, such as ultracentrifugation (UC), density gradient ultracentrifugation, size-exclusive chromatography (SEC), immunoaffinity, ultrafiltration, coprecipitation (polyethylene glycol-based) and newly developed microfluidics (46), and each method has its advantages and shortcomings. For example, UC, including differential centrifugation and sucrose density gradient ultracentrifugation, remains the most widely used method which relies on the size and/or density of EVs. However, the production efficiency of UC is limited by long time consumption, operator dependence and rupture of EVs (47, 48). Methods based on EV size, such as SEC, can improve the purity and stability of EVs, while EV yield is limited (49, 50). The immunoaffinity method can selectively enrich EVs with high purity, but it is unsuitable for large volume samples due to its high cost (51). For future clinical applications, a large amount of therapeutic EVs with high purity is needed, and thus, a major issue in this field is how to overcome the current problems of high cost and low yield for larger-scale production of EVs.

According to the minimal information for studies of extracellular vesicles (MISEV) guidelines, isolated EVs should be characterized at least by morphology, particle size and marker proteins (52, 53). The morphology of EVs is commonly observed using transmission electron microscopy or scanning electron microscopy (53). The particle sizes and numbers of EVs can be measured using nanoparticle analysis technology or dynamic light scattering (54, 55). Some key proteins involved in EV biogenesis or function, such as tetraspanins (e.g., CD9, CD63, CD81, CD82) or Alix, heat shock protein70 (HSP70), TSG101, and syntenin-1 associated with ESCRT, have been proposed as common EV markers (56, 57). However, the truth is that there are no specific markers for the identification of all EV subtypes until now (39). More importantly, for therapeutic purposes, the possible biosafety issues of EVs should be carefully considered, since it has been reported that some EVs from immortalized cell lines may carry oncogenic materials or endotoxins, while some EVs from infected cells may carry viral-derived proteins (34, 58). Thus, it is necessary to establish standardized and reproductive operating procedures, quality control criteria, and strictly sterile conditions during EV production (59).

### 3.4 Large-scale production and EV stability

For future clinical translation, a good manufacturing practice (GMP)-compliant protocol for EV production should be developed. The manufacturing of EV products can be briefly divided into upstream and downstream processings and subsequent quality control (60). In upstream processing, the large-scale expansion of EV-producing cells mainly depends on bioreactors, such as hollow fiber and stirred suspension bioreactors (using microcarriers) (61). Hollow fibers culture the cells in the hollow and semipermeable fibers and has less risk of contamination, while microcarriers are tiny beads with many varying features and require changing of culture media frequently (62). A recent study reported a turbulence strategy integrated into the cell culture in a stirred tank incompatible with a GMP bioreactor, which could obtain ~10^13^ EV particles from the supernatant of the one-liter bioreactor (63). In downstream processing, traditional isolation methods are not suitable for large-volume samples. Among the different methods, tangential flow filtration (TFF) may be one of the most powerful tools for industrial-scale EV manufacture (64) since it has a superior yield and bioactivity of isolated EVs compared to UC (65). Furthermore, TFF can be combined with other isolation approaches, such as SEC, to achieve higher scalability and reproducibility (66).

Stability is an important parameter for biological reagents, and the lipid bilayer structure of EVs provides unique protection to their bioactive cargoes (67). Some storage techniques that can long-term preserve EV bioactivities, such as freezing, lyophilization and spray-drying, have also been developed (68). For example, EVs suspended in buffered saline solution are stable for up to 2 years at -80°C without significant changes in properties (69), and the bioactivities of lyophilized EVs are similar to those of frozen EVs even stored at room temperature for 4 weeks (70). Therefore, the stability of EVs makes them ideal therapeutics in future clinical applications.

## 4 Therapeutic potential of native EVs

**Figure 4:**
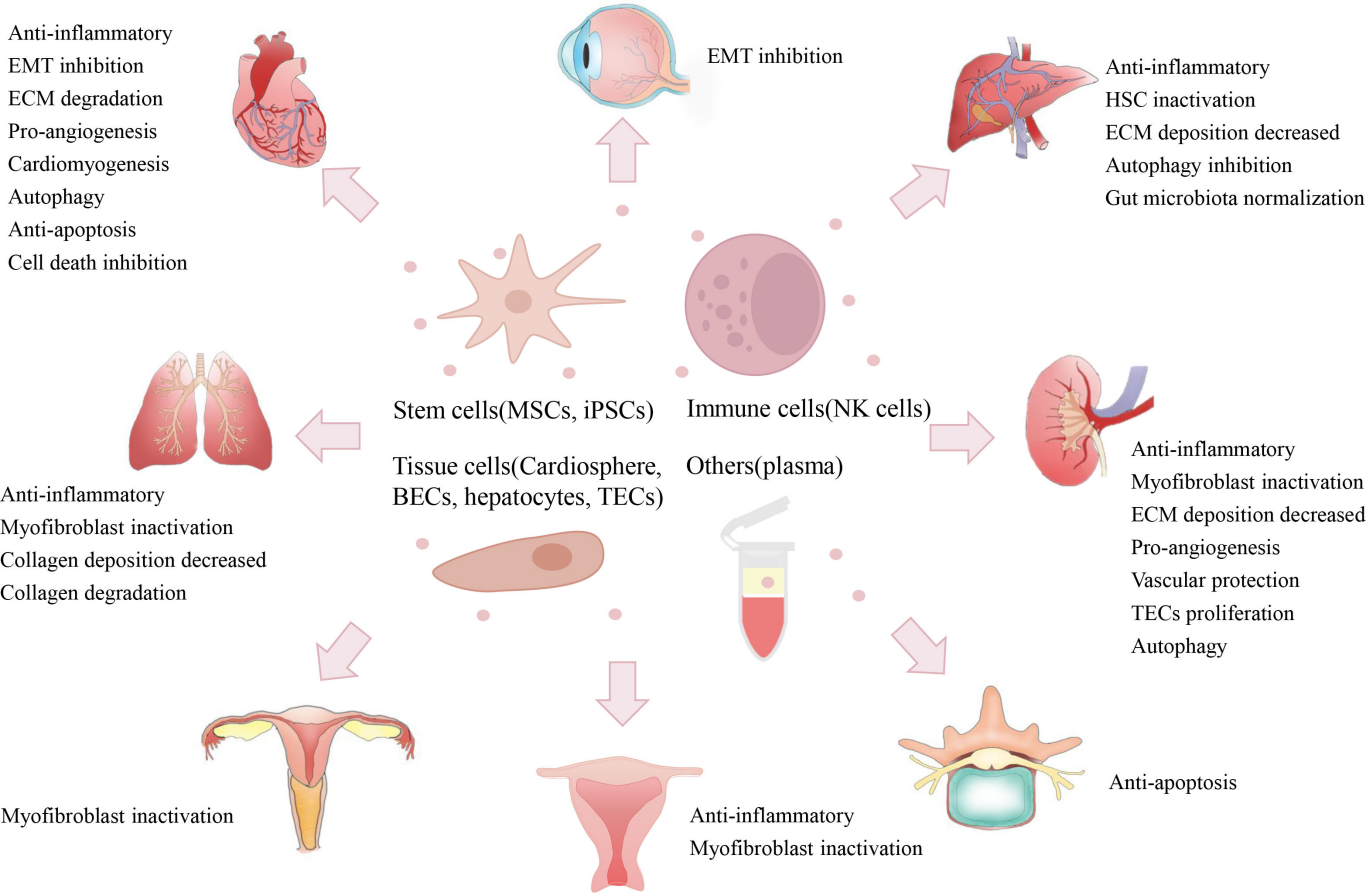
Therapeutic potentials of native EVs. Native EVs isolated from multiple cell sources (*e.g.*, stem cells, immune cells, tissue cells, and plasma) have exerted therapeutic efficacy on the alleviation of fibrosis in some major organs (*e.g.*, heart, lung, liver, and kidney) and other tissues (*e.g.*, IUA, IDD, and urethral stricture), and their therapeutic role may be due to multiple mechanisms, such as anti-inflammation, EMT inactivation, ECM degradation, and pro-angiogenesis. (Mesenchymal stem cells, MSCs; iPSCs, induced pluripotent stem cells; natural killer, NK; bronchial epithelial cell, BECs; tubular epithelial cell, TECs; epithelial to mesenchymal transition, EMT; extracellular matrix, ECM; intrauterine adhesion, IUA; intervertebral disc degeneration, IDD).

In recent decades, increasing numbers of studies have reported the potential of native EVs in preventing fibrosis *via* multiple mechanisms (**Supplementary Table 1**). Here, we briefly introduce the important studies regarding the anti-fibrotic effects (in major organs) of native EVs from various cell sources, such as stem cells, immune cells, tissue cells and blood (**Figure 4**).

### 4.1 Cardiac fibrosis

Cardiovascular disease (CVD) is one of the leading causes of death globally, and more than 17 million deaths worldwide are due to CVD per year (109, 110). Cardiac fibrosis can occur after almost all types of heart diseases, such as myocardial infarction (MI), hypertrophic cardiomyopathy (HCM), dilated cardiomyopathy (DCM), aortic stenosis (AOS), and diabetic cardiomyopathy (111, 112). Although some potential medicines, such as angiotensin (AT)-converting enzyme and angiotensin II receptor antagonists, β-blockers, endothelin antagonists, and statins have been used in the clinic to protect cardiac function, their direct antifibrotic efficacy remains debated (113, 114). Thus, new and advanced antifibrotic therapies are urgently needed.

Increasing evidence shows that native EVs isolated from stem cells may have the potential to prevent cardiac fibrosis (**Figure 4**). Mesenchymal stem cells (MSCs) are a type of adult stem cell with immunoregulatory and tissue repair potentials that can be isolated from multiple tissue types, such as bone marrow (BMSCs), umbilical cord tissue (ucMSCs) and adipose tissue (ADMSCs) (115). The possible antifibrotic effects of MSC-derived EVs (MSC-EVs) have been reported in multiple models of cardiac damage (such as hypertension and MI), and the underlying protective mechanism is linked to the inhibition of EMT (miR-200, *etc.*), anti-apoptosis, induction of M2 macrophages or pro-angiogenesis (**Supplementary Table 1**) (71, 72, 116, 117). Induced pluripotent stem cells (iPSCs) are a new source of embryonic-like stem cells obtained by reprogramming of somatic cells (*e.g.*, skin fibroblasts), which can further differentiate into many different cell types. iPSC-derived EVs reduced ECM deposition in the aortas of aged mice by inhibiting MMPs and elastase activity while enhancing the endothelial nitric oxide synthase (eNOS) pathway (73). Another therapeutic strategy is using EVs produced by iPSC-derived cardiac cells. For instance, iPSCs were first differentiated into contractile cardiomyocytes (iCMs), then iCM-derived EVs reduced the fibrotic areas of the left ventricle (LV) by enhancing autophagic flux after ischemic heart injury (74). EVs isolated from human embryonic stem cell (ESC)-derived cardiovascular progenitor cells recovered heart function (LV ejection fraction values/LV systolic dimensions) and reduced fibrotic areas after MI by promoting angiogenesis and inhibiting cardiomyocyte death (75).

In addition, native EVs from heart tissue cells also displayed certain antifibrotic effects in cardiac injury models (**Figure 4**; **Supplementary Table 1**) (118). The cardiosphere is a cluster of endogenous cardiac stem cells that forms when they are cloned in suspension, and cardiosphere-derived cells (CDCs) have been shown to have regenerative potential in cardiac injuries such as MI (119). CDC-EV treatment was shown to reduce the levels of cardiac hypertrophy, inflammation, and interstitial cardiac fibrosis in an Ang II-induced hypertension model by delivering Y RNA fragments to induce IL-10 expression (76). DCM is a serious pediatric cardiomyopathy, and ~50~60% of children with DCM will die in 5 years (120). In a swine model of DCM, CDC-EV treatment decreased myocardial fibrosis by shedding proangiogenic and cardioprotective miR-146a-5p to suppress inflammation (77). Regeneration-associated cells (RACs) are a group of heterogeneous cells (*e.g*., endothelial progenitor cells, M2 macrophages and regulatory T cells) under vasculogenic conditions after heart injury (121). RACs-derived EVs could reduce interstitial fibrosis after myocardial ischemic injury by shedding functional miRNAs (*e.g.*, miR-150-5p, miR-195 and miR-142-3p) to promote angiogenesis and cardiomyogenesis while reducing the inflammatory response (78). Altogether, these findings indicate that native EVs from multiple cell types may mitigate cardiac fibrosis *via* immunological regulation, pro-angiogenesis or suppression of cell death.

### 4.2 Lung fibrosis

Chronic lung diseases are highly associated with progressive lung fibrosis, resulting in poor quality of life and high mortality of patients (122). For example, IPF, an aggressive lung disease with an uncertain cause, has a poor prognosis with a median survival time of ~2-5 years after diagnosis (123) and ∼3 million patients worldwide suffer from IPF (124). However, the therapeutic efficacies of current treatments are not ideal in the clinic (125). For example, IPF patients receiving pirfenidone still have a high mortality rate and uncertain survival times (126). Therefore, advanced treatments for the resolution of lung fibrosis are also needed.

Native EVs from stem cells (*e.g.*, MSCs) have shown therapeutic potency in preclinical models of lung injury, and immunoregulation may be one of the main mechanisms involved (**Supplementary Table 1**). For example, BMSC-EV treatment attenuated profibrotic factor (TGF-β1, α-SMA, collagen I/III) expressions as well as fibrotic areas after lipopolysaccharide-induced acute lung injury, and this effect may be due to the inhibitory role of miRNAs (miR-23a-3p and miR-182-5p) on the NF-κB/Hedgehog pathways (79). In a silicosis model, ADMSC-EV intervention reduced the collagen contents and F4/80^+^ macrophage numbers and suppressed NF-κB/TLR activation in lungs by delivering functional miRNAs (*e.g.*, miR-146b) (80). In addition, the direct impact of MSC-EVs on profibrotic pathways had been reported. In a bleomycin (BLM)-induced IPF model, hucMSC-EV treatment inhibited myofibroblast differentiation and collagen deposition by delivering miR-21-5p/miR-23-3p to suppress the TGF-β pathway (81). BMSC-EV treatments also ameliorated fibroblast activation and α-SMA/collagen I expression in a BLM model, and miR-186 of EVs may inhibit SRY-related HMG box transcription factor 4 (SOX4), a regulator of lung development and monocyte infiltration (82, 83).

In addition, EVs from healthy lung tissue cells may be a possible therapy for the treatment of lung fibrosis (**Supplementary Table 1**). Airway epithelial cells (AECs) play regulatory roles in the development of lung fibrosis, and damaged bronchial epithelial cell (HBEC)-derived EVs can induce myofibroblast differentiation associated with airway remodeling (127). However, healthy AEC-EVs may have the opposite anti‐fibrotic effects. A recent study found that EVs from healthy HBECs could reduce TGF-β-induced myofibroblast differentiation *in vitro*, and intratracheal administration of these EVs promoted collagen degradation in BLM-induced IPF by inhibiting the crosstalks between the TGF-β and Wnt pathways (84). Lung spheroid cells (LSCs), an intrinsic source of lung stem cells, have been shown to mitigate fibrosis development in a rat BLM model (128). Similarly, human LSC-derived EVs (hLSC-EVs) also inhibited myofibroblast proliferation and collagen production by shedding miRNAs (miR-30a, miR-99 and let-7), and the inhalation of these EVs decreased BLM- or silica-induced rat lung fibrosis (85), suggesting that inhalation of EVs is a promising route for treating lung fibrosis. Altogether, these reports suggest that stem cells and healthy lung tissue cells are potential EV sources for mitigating lung fibrosis with inflammation and myofibroblasts as the possible targets.

### 4.3 Liver fibrosis

Chronic liver diseases are also a major health issue globally, with more than 800 million patients affected and ~2 million deaths per year (2). Liver fibrosis can be triggered by many causes, such as alcohol, nonalcoholic hepatic steatohepatitis (NASH), and biliary atresia, and the fibrosis is associated with cirrhosis, liver failure and portal hypertension (11). Current clinical therapies for patients with chronic liver diseases are including the removal of the pathological cause, management of the complications, and final liver transplantation (11, 129). However, these strategies are not efficient due to the continued growth of cirrhosis and the paucity of available donor livers (130). Thus, the development of novel treatments to lighten the burden of patients with liver fibrosis is necessary.

Native EVs from MSCs and iPSCs are also major players in studies related to liver fibrosis therapy (**Supplementary Table 1**). For instance, human amnion MSC-derived EV treatment reduced α-SMA expression and fibrotic areas in a rat CCl_4_-induced liver fibrosis model by inhibiting Kupffer cells and hepatic stellate cells (HSCs) activations (86). Chemokines, such as CXC chemokine ligands (CXCLs), are involved in the activation of HSCs through autocrine and fibrogenesis (131). Allogenic ADMSC-EVs reduced the CCl_4_-induced collagen volume fraction and α-SMA/collagen I/III expression by transferring miR-150-5p to inhibit CXCL1 signaling in a mouse CCl_4_ model (87). Chronic graft-versus-host disease (cGVHD) is a common complication of allogeneic hematopoietic stem cell transplantation and is highly associated with major organ damage, such as liver damage (132, 133). In cGVHD mice, hBMSC-EVs alleviated the degree of liver fibrosis and prolonged animal survival by inducing IL-10^+^ regulatory cells and inhibiting IL-17^+^ pathogenic T cells (88). In another study, human skin fibroblast-derived iPSC-secreted EVs decreased HSC activation and liver fibrotic areas by shuttling functional miRNAs (*e.g.*, miR-92a-3p) in a CCl_4-_ or bile duct ligation-induced mouse model (89).

In addition, EVs from liver or other tissue-derived stem cells also showed potential anti-fibrotic effects (**Supplementary Table 1**). Human liver stem cells (hLSCs) are a stem cell population derived from human adult liver cells that exhibit antifibrotic effects in a murine model of NASH (134). A recent study found that hLSC-derived EVs could attenuate plasma alanine aminotransferase (ALT) and liver fibrosis in a murine model of NASH by delivering miRNAs (miR-29a, miR-30a and let-7) to inhibit collagen I and Snail expression (90). Tonsils are an alternative source of MSCs since tonsil-derived MSCs (T-MSCs) can be readily obtained from surgically removed tonsil tissues. T-MSC-EV treatment reduced the expression of α-SMA, TGF-β, Vimentin and connective tissue growth factor (CTGF) in the liver of the CCl_4_ model by delivering miR-486-5p to inhibit Hedgehog signaling (91).

Interestingly, EVs from immune cells or healthy liver cells may have a potential role in preventing liver fibrosis (**Supplementary Table 1**). As a type of innate immune cell, natural killer (NK) cells are important regulators of HSC activation (135, 136). NK-cell line (NK-92MI)-derived EVs reduce TGF-β-induced HSC activation and α-SMA/collagen expression by transferring miR-223 to inhibit the autophagy-related (ATG7) pathway (92). EVs from primary human hepatocytes could also attenuate CCl_4_-induced hepatocyte injury and profibrotic factor (α-SMA, collagen and CTGF) expression (93). In addition to eukaryotes, EVs isolated from prokaryotes may also have antifibrotic effects. *Akkermansia muciniphila* is a probiotic with beneficial effects on the host metabolic system and immune response. In a high-fat diet (HFD)- and CCl_4_-induced mouse liver injury model, probiotic-derived EVs reduced liver functional damage and fibrotic factor (TGF-β, α-SMA and collagen I) expressions by normalizing gut microbiota composition disorders (94).

### 4.4 Renal fibrosis

Chronic kidney disease (CKD) is a huge public health issue that attributed to ~1.5% of deaths worldwide in 2012 (3). Renal fibrosis is the key feature of CKD and the leading cause of the end-stage renal disease (ESRD) and renal failure (137). Current clinical CKD therapeutic strategies mainly focus on controlling ongoing nephron injury, hyperfiltration and renal complications. Once CKD patients progress to ESRD, the only treatment is kidney replacement therapy or kidney transplantation (138). Thus, more efficient therapeutics to prevent renal fibrosis are needed.

As shown in **Supplementary Table 1**, EVs isolated from stem cells remain as a major candidate for antifibrotic therapy in kidneys (139). For example, hBMSC-EV treatment reduced α-SMA and ECM (collagen I) expressions in a mouse aristolochic acid nephropathy (AAN) model (95). Yes-associated protein (YAP) plays a vital role in fibrogenesis by retaining activated Smad2/3 in the cell nucleus (140). EVs from hucMSCs alleviated interstitial fibrosis by promoting YAP degradation in a rat UUO model (96). Autophagy is a conserved cellular process that removes unnecessary or damaged components, while it may be impaired in the diabetic nephropathy (DN) state (141). In a rat DN model, BMSC-EVs were found to reduce collagen accumulation by inducing mTOR-mediated autophagy (97). Importantly, the antifibrotic effects of MSC-EVs can be replicated in large animal models. In a porcine model of metabolic syndrome (MetS), the systemic administration of ADMSC-EVs restored kidney function and reduced tubulointerstitial fibrosis by IL-10-dependent immunoregulation (98). Additionally, intrarenal injection of ADMSC-EVs decreased ECM expression in another swine MetS model by inducing regulatory T cells (99). These findings suggest that *in situ* administration of EVs may be an efficient way to improve their therapeutic potency.

Renovascular disease (RVD), represented by the narrowing of one or both renal arteries, can cause high blood pressure and renal dysfunction in patients. In a swine atherosclerotic RVD model, intrarenal-injected ADMSC-EVs decreased interstitial fibrosis while improving the glomerular filtration rate (GFR) and peritubular capillary density by shedding some vasculo-protective genes (*e.g.*, HGF and VEGFR) (100). In addition, EVs isolated from other stem cells may also have therapeutic effects. For example, mouse ESC-derived EVs reduced renal dysfunction (creatinine and blood urea nitrogen) and α-SMA expression by promoting tubular epithelial cell (TEC) proliferation and angiogenesis in a mouse model of ischemic acute kidney injury (AKI) (101). In a mouse AAN, hLSC-derived EVs reduced renal interstitial fibrosis *via* miR-29b-mediated inhibition of the Wnt/β-catenin pathway (102).

In addition to stem cells, EVs from healthy kidney tissue cells may also have an antifibrotic role (**Supplementary Table 1**). For example, in a rat ischemic AKI model, renal tubular cell-derived EV treatment reduced collagen I/II/IV/V deposition, α-SMA/FN expression and neutrophil infiltration by shuttling mRNAs encoding cytoplasmic ribosomal proteins (Rps6 and Rps13) (103). Interestingly, EVs isolated from the plasma of MI could inhibit the apoptosis and autophagy of TECs (NRK-52E) *in vitro*, and decrease the fibrotic area in a contrast-induced nephropathy (CIN) model by delivering miR-1-3p to target ATG13 and activate the AKT pathway (104). These studies suggest the potential of native EVs in attenuating renal fibrosis through a variety of mechanisms.

### 4.5 Other organ fibrosis

In addition to these major organs, many other organs or tissues can also undergo fibrosis (**Figure 4**). For example, in the reproductive system, intrauterine adhesion (IUA) is a serious fibrotic disease due to disordered repair after endometrial basal layer damage. Rabbit BMSC-derived EV treatment inhibited endometrial fibrosis and the TGF-β1/Smad2 pathway in a rabbit model of endometrial injury (105). Subretinal fibrosis is a common complication of macular neovascularization and causes irreversible loss of central vision (142). hucMSC-EV treatment was found to suppress the EMT of retinal pigment epithelial cells *in vitro* and reduce laser-induced subretinal fibrosis in mice by shedding miR-27-3p to inhibit the homeobox protein Hox-C6 (HOXC6) (106). Intervertebral disc degeneration (IDD) is a major cause of chronic discogenic back pain/sciatica which results in poor quality of life in patients. Fibrosis progression of nucleus pulposus (NP) cells plays a vital role in the pathology of IDD. Rat BMSC-EVs reduced TNF-α-induced ECM (collagen I) expression in NP cells by inhibiting proapoptotic RASSF5 signaling *via* miR-532-5p (107). Urethral stricture (abnormal narrowing of the urethra) is a complication of urological surgery and is caused by fibrosis of the urethral epithelium due to damage or infection. In a rat urethral injury model, hucMSC-EV treatment inhibited myofibroblast activation by transferring anti-inflammatory miR-146a (108). These reports indicate that MSC-EVs may exert antifibrotic effects in many types of organs by inhibiting EMT, inflammation or apoptosis.

## 5 Engineering strategies of EV-based anti-fibrotic therapies

**Figure 5:**
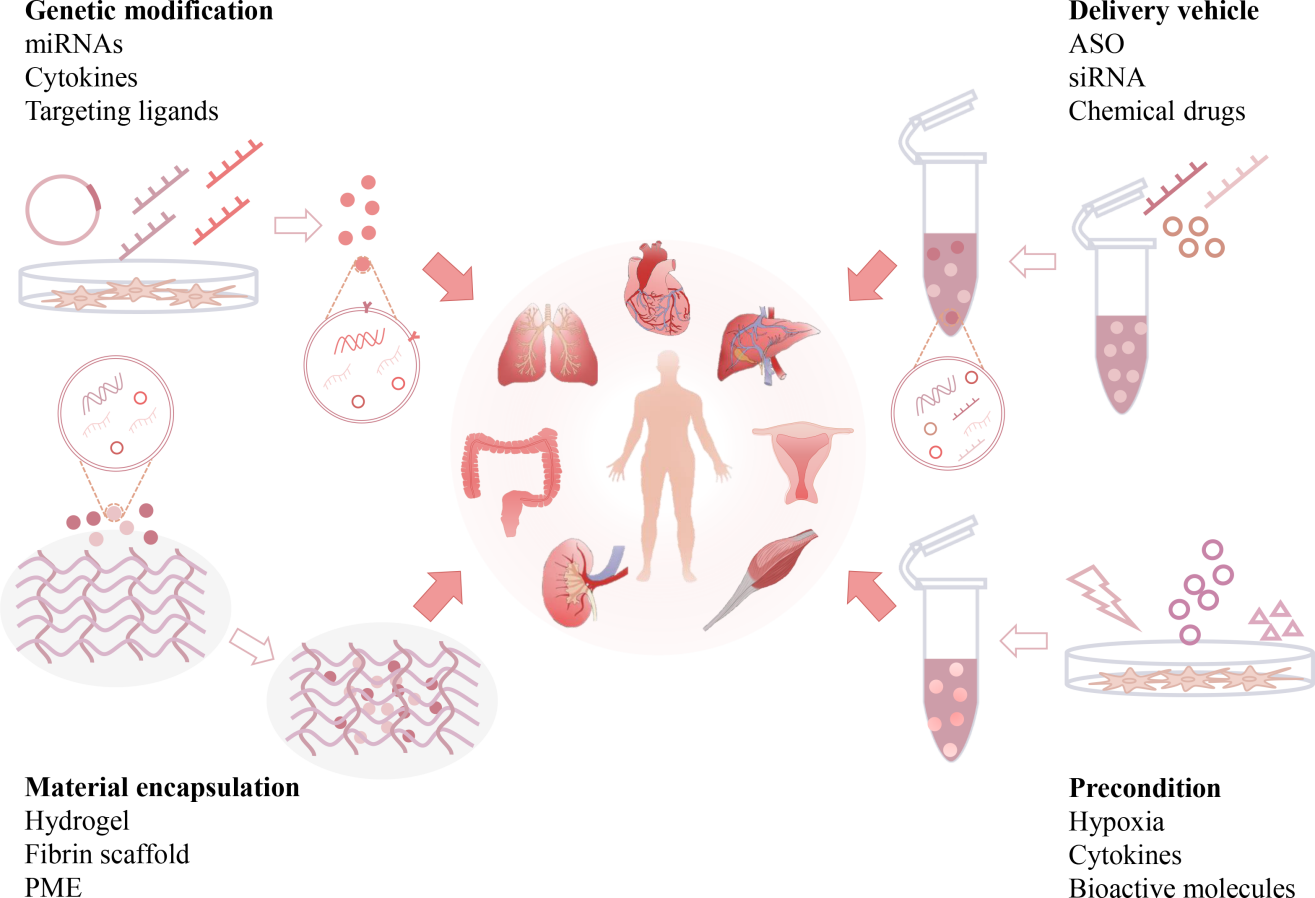
Engineering strategies of EV-based anti-fibrotic therapies. Native EVs can be re-engineered by multiple strategies, such as genetic modifications, preconditions, loading with therapeutic reagents, and incorporation with functional materials. Engineered EVs can exhibit additional beneficial effects, such as enhanced drug payload, longer half-life, better organ targeting capability and bioavailability, and serve as advanced nanomedicines for the resolution of organ fibrosis. (Antisense oligonucleotide: ASO, pneumatic microextrusion: PME).

Although native EVs isolated from multiple cell types have shown certain antifibrotic effects, their therapeutic efficiency may be restricted by some intrinsic limitations, such as short half-life, low specific organ retention, possible off-target effects, and insufficient cell sources. Therefore, the development of engineered EVs may resolve these issues and enhance their therapeutic potency (143). Abundant evidence shows that native EVs can be re-engineered by biological or chemical methods and serve as advanced nanomedicines for the resolution of organ fibrosis. In the following sections, we briefly discuss the current modification strategies and therapeutic outcomes of engineered EVs in different organ fibrosis models (**Figure 5** and **Table 2**).

**Table 2:** Therapeutic effects of engineered EVs.

Modifications	Fibrotic models	EV origins	Isolation methods	Reengineering routes	Therapeutic effects	Possible mechanisms	ref.
Genetic engineering	CCl_4_	mice ADMSC	ExoQuick-TC Kit	Transfection to overexpress miRNA-181-5p	*In vivo:* AST, ALT, TB↓ COL I, Vimentin↓ Inflammation↓ *In vitro*: COL I, COL III, FN, α-SMA, Vimentin↓	Inhibiting the apoptosis pathway	(144)
	tendon adhesion	hucMSC	UC	Using antagonist to underexpress miR-21a-3p	*In vivo:* Tendon adhesion↓ α-SMA, COL III↓ *In vitro*: Fibroblast proliferation↓ α-SMA, COL III↓	Inhibiting NF-κB activity	(145)
	UUO	human ADMSC	UC	Lentiviral transfection to overexpress GDNF	*In vivo*: Interstitial area↓ Perfused capillaries↑ α-SMA↓ *In vitro*: Migration↑ Tube formation↑	Pro-angiogenesis	(146)
	MI	hucMSC	Total Exosome Isolation reagent	Using CRISPR/Cas9 to silence β-2 microglobulin	*In vivo*: Fibrotic area↓	Preventing the immune rejection	(147)
	UUO	Primary mouse satellite cells	UC	Using Lamp2b fused with RVG	*In vivo:* α-SMA, COL 1A1, COL 4A1, Vimentin, FN↓ myoD, myogenin, eMyHC↑	Downregulation of TGF-β pathway	(148)
Delivery vehicle	CCl_4_	human BMSC	UC	Carrying siRNA or ASO targeting STAT3	*In vivo:* α-SMA, COL I, Vimentin, FN, Col 1a1↓	Anti-inflammatory	(149)
	Adriamycin	RAW 264.7	centrifugation	Delivery DEX	*In vivo:* Interstitial fibrosis↓	Anti-inflammation	(150)
Precondition	MI	mice BMSC	UC	Hypoxia condition (0.5% O_2_) and culture for 24 h	*In vivo:* Cardiac function↑ Fibrotic scar size↓	Delivery of anti-apoptosis miR-210	(151)
	Urethral stricture	hucMSC	UC	Pretreatment with 10ng/mL TNF-α for 12h	*In vivo:* α-SMA↓ Collagen fibers↓ *In vitro:* Fibroblast activation↓ α-SMA, COL I, COL III↓ IL-6, IL-1β↓	Delivery anti-inflammatory miR‐146a	(108)
	Sulfate-Induced Colitis	mice BMSC	Ultrafiltration	Pretreatment with IL-1β (25 ng/mL), IL-6(20 ng/mL) and TNF-α (20 ng/mL) for 24h	*In vivo:* Collagen deposition↓ Necrotic mucosal surface↓	Anti-inflammatory	(152)
	CCL_4_	RAW264.7	sequential centrifugation	Pretreatment with 100ng/mL RLN for 24h	*In vivo:* Serum AST, ALT↓ Hepatic hydroxyproline↓ α-SMA↓ *In vitro:* α-SMA, COL Iβ↓	Anti-inflammatory	(153)
Material encapsulation	Ischemic AKI	mice BMSC	UC	KMP2 hydrogel to release EVs	*In vivo*: BUN, CREA↓ NGAL↓ α-SMA, FN↓ Inflammation↓ Renal microvascular injury↓	Decreasing cell apoptosis/inflammation and improving microvascular endothelial cell regeneration	(154)
	Thioacetamide induce chronic liver injury	human ESC	UC	EVs embedded with a clickable PEG	*In vivo*: Hepatoprotective effects↑ COL I, α-SMA↓ MMP-9, MMP-13↑		(155)
	MI	MSC	ultrafiltration	Seeding EVs in fibrin scaffold	*In vivo*: Infarct size↓ LV wall↑ Viable cardiac tissue↑	Promoting endogenous angiomyogenesis	(156)
Combination	partial nephrectomy	hucMSC	UC	A hybrid scaffold of PME and PDRN combined with MSC-EVs primed with 20 ng/mL IFN-γ and TNF-α for 72h	*In vivo:* CREA, BUN↓ GFR↑ α-SMA, Vimentin, Snail1↓ IL-1RA, TNF-α↓ IL-10↑ *In vitro:* N-Cadherin, FN↓		(157)
	BLM	L-929	UC	Hybrid nanovesicles to delivery NIN	*In vivo:* Pulmonary function↑ Collagen deposition↓ α-SMA, MMP-7, TGF-β↓	Diminishing macrophage-induced inflammatory response	(158)
	Sulfate-Induced Colitis	Human dental pulp MSCs	UC	HIF-1α overexpression and TNF-α (10 ng/mL), IL-1β (10 ng/mL) and IFN-γ (50 ng/mL) pretreatment	*In vivo:* Fibrillar collagen proportion↓ *In vitro:* α-SMA, COL 1α↓		(159)

GDNF, glial cell line-derived neurotrophic factor; CRISPR/Cas9, clustered regularly interspaced short palindromic repeats/CRISPR-associated endonuclease; RVG, rabies viral glycoprotein peptide; ASO, antisense oligonucleotide; DEX, dexamethasone;TNF-α, tumor necrosis factor-α; RLN, relaxin; PEG, polyethylene glycol; PME, pneumatic microextrusion; PDRN, polydeoxyribonucleotide; IFN-γ, Interferon-γ; NIN, nintedanib.

### 5.1 Engineered EVs as anti-fibrotic agents

#### 5.1.1 Genetic modifications

Genetic engineering of parental cells is a common strategy to produce functional EVs. Engineered EVs with enhanced therapeutic properties or organ targeting efficacy can be obtained from donor cells with genetic modifications, such as gene or protein (*e.g.*, miRNAs and cytokines) overexpression or knockdown (7). Vector transfection and transduction are the main methods used for the genetic engineering of EV donor cells (66). For example, mouse ADMSC-derived EVs were engineered to overexpress miRNA-181-5p, a regulator of hepatic progenitor cell differentiation and autophagy (144). As a result, these engineered EVs had high potency to rescue liver function and reduce ECM (collagen I and FN) and vimentin expression in a CCl_4-_induced liver fibrosis model (144). miRNA-21a-5p can synchronize with NF-κB activation and it is abundant in fibrotic tissues. Engineered hucMSC-EVs with antagomir-21a-5p showed higher potential in inhibiting fibroblast activation *in vitro* and α-SMA and collagen III expression in a rat tendon adhesion model than native EVs (145).

EVs can also be engineered with therapeutic proteins (*e.g.*, cytokines). Glial cell line-derived neurotrophic factor (GDNF) is a member of the TGF-β superfamily and plays a vital role in renal morphogenesis and angiogenesis (160). In a UUO renal fibrosis model, GDNF-overexpressing hADMSC-EVs exhibited higher potency to reduce peritubular capillary rarefaction and renal fibrosis score/α-SMA levels than native EVs (146). Disruption of the human leukocyte antigen (HLA) light chain β2-microglobulin (B2 M) gene may disable the function of HLA-I molecules and thus prevent hucMSC-mediated immune rejection (161). hucMSCs with B2 M deletion (B2 M^-^hucMSCs) were generated using a CRISPR/Cas9 method, and these modified EVs were more efficient in reducing fibrotic areas and restoring cardiac function than native EVs in a rat model of MI (147).

Furthermore, the organ targeting capability of native EVs can be improved by genetic modifications. Fusion of targeting ligands (*e.g.*, peptides) into EV membrane proteins (*e.g.*, Lamp2b and CD63) is a common strategy to produce EVs with specific cell- or tissue-targeting ability (162). For example, a recent study showed that engineered mouse satellite cell-derived EVs using Lamp2b fused with a rabies viral glycoprotein peptide (RVG) had higher renal targeting efficacy. In a UUO model, delivery of miR-29 (a potent inhibitor of TGF-β3) by these modified EVs had higher antifibrotic potency (reducing renal α-SMA, collagen, vimentin and FN levels) than unmodified EVs (148). Altogether, these studies indicate that genetic engineering is a potent strategy to enhance the organ targeting and therapeutic effects of EVs.

#### 5.1.2 Delivery of therapeutic reagents by EVs

Due to their bilayer structure and cargo transferring capacity, EVs can serve as natural carriers of therapeutic agents and protect them against *in vivo* degradation. The current techniques of loading cargoes into EVs can be divided into exogenous loading (coincubation, electroporation, extrusion or sonication of drugs with isolated EVs) and endogenous loading (genetic modification of EV donor cells) (66, 163). Antisense oligonucleotide (ASO)/siRNA is a type of nucleic acid drug that can silence targeted genes, but its therapeutic potency is largely limited by off-target effects and liver toxicity (164, 165). To overcome these limitations, ASO or siRNA targeting STAT3 (a key regulator of inflammation) was loaded into BMSC-EVs, and the engineered iExo^siRNA-STAT3^ or iExo^mASO-STAT3^ treatment showed higher potential to reduce collagen I deposition and α-SMA, vimentin and FN expression in a CCl_4_-induced liver fibrosis model (149).

In addition, EVs can also be used to deliver chemical drugs to enhance their therapeutic index *in vivo* (**Table 2**). The anti-inflammatory drug, dexamethasone (DEX), has been used for fibrosis treatment (166). In a recent study, RAW 264.7 macrophage-derived DEX-packaging EVs exhibited a superior capacity to suppress renal inflammation and interstitial fibrosis without apparent glucocorticoid adverse effects in Adriamycin-induced nephropathy in mice (150). These studies suggest that engineered EVs are potent carriers for the delivery of therapeutic agents to alleviate fibrosis.

#### 5.1.3 Preconditioned EVs

In addition to the mentioned EV modification methods, parental cells can also be primed/preconditioned with pathological stimuli to produce EVs with desirable profiles or contents useful for fibrosis resolution (**Table 2**). MSCs are usually preconditioned with hypoxia or cytokines to acquire and retain phenotypes relevant for therapeutic applications (167). For instance, EVs isolated from BMSCs under hypoxic conditions (0.5% O_2_ for 24 h) had superior therapeutic ability in restoring cardiac function and ameliorating fibrotic scar area after MI, because those EVs had enriched miRNAs such as the anti-apoptotic miR-210 (151). Pretreatment of donor cells with cytokines can augment the immunomodulatory properties of the produced EVs. For example, TNF-α-preconditioned hucMSC-EVs reduced α-SMA and collagen expression in a rat urethral fibrosis model due to the enriched anti-inflammatory miR-146a in those EVs (108). EVs from mouse BMSCs primed with a cocktail of cytokines (IL-1β, IL-6 and TNF-α) had higher efficacy in reducing the necrotic mucosal surface and collagen deposition in a dextran sulfate sodium-induced colitis model (152).

In addition, pretreatment of donor cells with bioactive molecules may also enhance the therapeutic effects of EVs (**Table 2**). Relaxin (RLN) is an antifibrotic peptide hormone that has been shown to reduce liver fibrosis by reversing the activation of HSCs (168). In a mouse CCl_4_ liver fibrosis model, EVs from RLN-preconditioned mouse macrophage cell lines (Raw264.7) showed higher potency to lower serum ALT/aspartate aminotransferase (AST) and reduced liver fibrosis (hydroxyproline and α-SMA levels) than native EVs (153). These studies suggest that the preconditioning of parental cells may be an efficient method to produce functionalized EVs.

### 5.2 Biomaterials for EV retention and delivery

Current evidence indicates that systemically administered EVs have a short half-life and can be rapidly cleared *in vivo*. To resolve this problem, an innovative strategy that encapsulates EVs with functional biomaterials has been proposed (**Figure 5** and **Table 2**). EVs delivered by biomaterials such as hydrogels and scaffolds may have enhanced therapeutic efficacy due to prolonged EV release and improved bioavailability (169). Hydrogels are three-dimensional hydrophilic networks with advanced properties, such as tunability, good biocompatibility and biodegradability, and high tissue retention, therefore, they have been widely used to encapsulate cells or drugs. This strategy can also be applied in the local delivery of EVs to improve their stability and half-life (170). Self-assembling peptide (SAP) is a type of biomaterial made of natural amino acids, and some of them can rapidly form a nanoscale hydrogel in ionic saline conditions (171). An injectable SAP hydrogel was used to encapsulate BMSC-EVs, which enabled a sustained release of EVs with preserved biofunction. In a mouse model of ischemic AKI, local delivery of BMSC-EVs by SAP hydrogels showed better efficacy in decreasing renal damage, inflammation and subsequent renal fibrosis (α-SMA and FN) than EVs alone (154).

Similarly, human ESC-EVs were encapsulated into a polyethylene glycol (PEG) hydrogel through a click reaction. The resulting EV-loaded hydrogel showed higher antifibrotic effects in a thioacetamide-induced liver fibrosis model, as indicated by lower expression of MMP-9/13, collagen I and α-SMA than EVs alone (155). The fibrin scaffold is a degradable biopolymer made of fibrinogen and can provide binding sites for cell migration and proliferation to promote tissue regeneration (172). In another study, an invasive EV spray was prepared by incorporating MSC-EVs with fibrin scaffold materials. In a mouse or a swine model of MI, EV spray treatment led to smaller infarct size, thicker left ventricular wall, and enhanced angiomyogenesis than EVs alone in the postinjury heart (156). Altogether, these findings suggest that biomaterial-based EV engineering is an efficient strategy for enhanced antifibrotic therapy.

### 5.3 Combined strategies for enhancing therapeutic potency

Moreover, the combination of multiple EV modifications may be an attractive strategy for advanced anti-fibrotic therapy (**Table 2**). For example, cytokine (TNF-α and IFN-γ)-preconditioned hucMSC-EVs were loaded into a hybrid scaffold made of pneumatic microextrusion (PME, consisting of PLGA, magnesium hydroxide and decellularized porcine kidney extracellular matrix) and polydeoxyribonucleotide (PDRN). This combined treatment showed a synergistic effect of reducing renal inflammation (IL-1RA and TNF-α) and fibrosis (α-SMA, vimentin and Snail) in a mouse model of partial nephrectomy (157). NIN is a tyrosine kinase receptor inhibitor for the treatment of lung fibrosis in the clinic, but its therapeutic efficiency is not ideal due to nonspecific organ distribution. In a recent study, NIN was loaded into hybrid nanovesicles made of clodronate disodium (CLD)-loaded liposomes and fibroblast cell line (L-929)-derived EVs. In a BLM-induced mouse lung fibrosis model, this engineered hybrid EVs showed higher lung retention than unmodified EVs and thus decreased macrophage-mediated inflammation and ECM deposition in the lungs (158). In addition, the combination of stimuli and genetic modification may enhance the therapeutic potency of native EVs. For example, EVs from human dental pulp MSCs with HIF-1α (a master regulator of hypoxia) overexpression and cytokine (IFN-γ, IL-1β and TNF-α) preconditioning were produced, and these EVs had higher potency to alleviate the fibrillar collagen proportion and colon length shortening in a mouse colitis model than native EVs (159). Altogether, the combination of multiple engineering strategies may further enhance the anti-fibrotic potency of EV-based therapies.

## 6 Clinical trials of EVs for fibrosis-related diseases

To date, few clinical trials have been conducted associated with direct organ fibrosis, and most of them are still in the early stages. For example, a randomized, placebo-controlled, phase 2/3 clinical pilot study was performed to investigate the safety and therapeutic efficacy of cord-blood MSC-EVs in preventing the progression of grade III and IV CKD. The results showed that intra-arterial and intravenous EV injections reduced the inflammatory immune reaction and improved kidney function (173). Recent studies suggest that severe acute respiratory syndrome coronavirus 2 (SARS-CoV-2) infection may cause severe lung damage and substantial lung fibrotic consequences in patients (174). To protect the damaged lungs, several EV-based clinical trials are already underway or seem to be underway. A multicenter, double-blind, randomized controlled trial (RCT) phase 2/3 trial is recruiting, and it aims to evaluate the efficacy and safety of MSC-EVs on reducing inflammation in moderate COVID-19 patients (NCT05216562). An open-label phase 1 study was conducted to evaluate the safety and immunoregulation of EVs carrying CD24 in patients with moderate/severe COVID-19 (NCT04747574). These modified EVs are produced from T-REx™-293 cells engineered to express CD24 (a vital immunomodulator), and its phase II trial is currently active, not recruiting (NCT04969172). Although the current state is still far from clinical applications, increasing evidence suggests that EVs are a potent cell-free, off-the-shelf antifibrotic strategy.

## 7 Future perspectives

Notably, a large number of studies indicate that EVs may be promising means to mitigate organ fibrosis. Moreover, as a type of naturally derived nanomaterial, EVs have several advantages, such as intrinsic biological properties, the ability to cross biological barriers, and minimal immunogenicity or toxicity *in vivo (* 7, 51). However, there are still some limitations that needed to be well resolved before further translation of EVs into clinical applications.

A primary challenge in this field is how to standardize the production of EVs on a large-scale during isolation, purification, and scalability. For research purposes, small amounts of EVs can be readily isolated by common techniques, such as UC, SEC and immunoaffinity (47), but the yields are far below the clinical requirement. For small animal models, such as rodents, the medium EV dosage is ~80-100 μg/mouse with systematic injection, while it dramatically increases to 75 mg/swine *in situ* in large animal models (175, 176). In this case, it can be assumed that the required EV amounts for human patients with fibrotic diseases are much larger. Thus, it is urgent to optimize the process of large-scale EV production with high yield and purity, as well as retain integrity and biofunction. More importantly, for clinical applications, it is strictly required to define the bioactive components, standard operating procedures, quality control criteria, virulence and sterility of EV products.

Native EVs usually exhibit a short half-life and insufficient organ retention *in vivo*. The distribution of EVs can be affected by many factors, such as parental cell types or administration routes (177). To improve the specific organ targeting efficiency of EVs, it is possible to select the appropriate cell source according to the therapeutic intent, such as bronchial epithelial cell-derived EVs for lung fibrosis and cardiosphere-derived EVs for cardiac fibrosis (77, 84). Another strategy is surface modification of EVs with targeting ligands, thereby enhancing the uptake of therapeutic EVs in targeted organs. The administration routes of EVs may also affect their therapeutic potency. For instance, i.v. injected EVs showed higher retention in the liver and spleen and lower retention in the pancreas compared to i.p. and subcutaneous injection (177). Thus, the selection of a proper delivery route should also be considered in the treatment of fibrosis in different organs.

Since the mechanism of fibrosis is complicated and many types of cells and pathways are involved, it seems that single-target therapy may not be efficient. In fact, many antifibrotic strategies, such as cessation of chronic tissue injury, resolution of local inflammation, deactivation or elimination of myofibroblasts, and degradation of ECM, have been evaluated individually or combined in preclinical studies or clinical trials (5). However, many current EV-based studies are still focusing on targeting a single cause (*e.g.*, inflammation and angiogenesis), and the direct antifibrotic potency of EVs needs to be further enhanced. To address this limitation, a better strategy is to develop engineered EVs that can target multiple essential cells or pathways involved in fibrosis, which may exhibit advanced therapeutic effects.

Another important limitation is that current findings related to the anti-fibrotic effects of EVs are mainly based on small animal (mouse and rat) models, while there have been only a small number of large animal (*e.g.*, swine) model-based studies (156). Moreover, these models may not fully mimic the pathology of human patients due to the large genetic and/or physiological differences between humans and small animals. For example, rodent models of liver fibrosis are commonly induced by toxic reagents (*e.g.*, CCl_4_) or bile duct ligation, while virus (hepatitis B virus) infection, alcoholic liver disease, and nonalcoholic fatty liver disease are the main causes of liver fibrosis in the clinic (2, 178). Thus, standard and large animal models that can better mimic the fibrosis of patients are needed in this field.

## 8 Conclusion

In summary, EV-based therapeutics have shown promising effects in mitigating multiple types of organ fibrosis in many preclinical studies. Moreover, the therapeutic potential of EVs can be further improved using multiple modification strategies. To better translate EV-based therapies into clinical applications, more research is required to clarify the direct antifibrotic role of EVs and to establish large-scale production and efficient EV engineering strategies in the future.

## Author contributions

KL wrote the manuscript and prepared for visualization. YW, PL, SL and PZ prepared for drawing elements, LY and YL played as supervision roles. JC provided financial support. JL is corresponding author and review the manuscript. All authors contributed to manuscript revision, read, and approved the submitted version. All authors contributed to the article and approved the submitted version.

## Funding

This study was partly supported by the National Natural Science Foundation of China (32071453, 31871001, 81571808, 32271438, 32001011), 1.3.5 Project for Disciplines of Excellence (ZYGD18014), the Fundamental Research Funds for the Central Universities (2020SCU12040), and the PostDoctor Research Project of West China Hospital of Sichuan University (2020HXBH047).

## Acknowledgments

We would like to thank Wenshu Dai from Sichuan University for language editing.

## Conflict of interest

The authors declare that the research was conducted in the absence of any commercial or financial relationships that could be construed as a potential conflict of interest.

## Publisher’s note

All claims expressed in this article are solely those of the authors and do not necessarily represent those of their affiliated organizations, or those of the publisher, the editors and the reviewers. Any product that may be evaluated in this article, or claim that may be made by its manufacturer, is not guaranteed or endorsed by the publisher.
