# Supplementary material for: Extracellular vesicles as advanced therapeutics for the resolution of organ fibrosis: Current progress and future perspectives
Source: Front Immunol. 2022 Oct 20;13:1042983. doi: 10.3389/fimmu.2022.1042983 (PMC9630482; doi:10.3389/fimmu.2022.1042983)
Supplement: Supplementary file 1 [file Table_1.docx]

**Supplementary Table 1. Therapeutic effects of native EV**

| **Organs** | | **Fibrotic models** | **EVs origins** | **Isolation method** | **EVs dosage/routes** | **Therapeutic effects** | **Possible mechanisms** | **ref.** |
| --- | --- | --- | --- | --- | --- | --- | --- | --- |
| **Heart** | | Deoxycorticosterone acetate | Human ADMSC | UC | *In vivo:*  1.5 × 10^9^/week for 9weeks | *In vivo:*  Fibrosis area↓ | Regulation of the axis miR-200-TGF-β | (78) |
|  | | MI | Rat ADMSC | UC | *In vivo:*  2.5 × 10^12^ particles  *In vitro:*  200 μg/ml | *In vivo:*  LV ejection fraction, LV fraction shortening↑  Fibrosis area↓  α-SMA, COL I, COL III↓  *In vitro:*  TGF-β↓ | Promoting macrophage M2 polarization | (79) |
|  | | Aging related hypertension | Human iPSC | UC | *In vivo:*  18 x 10^6^/mouse/injection per week for 4weeks | *In vivo:*  Arterial compliance↑  Endothelial dysfunction↓  Arterial elastin degradation↓  Elastase activity↓  COL I↓  TGF-β, MMP-2, MMP-9↓ | Activation of eNOS pathway and inhibition of MMPs and elastase | (82) |
|  | | MI | Human iPSC-CM | Polyethylene glycol 8000 | *In vivo:*  4×10^8^ | *In vivo:*  LV ejection fraction↑  Fibrosis area↓ | Enhancing autophagosome production and flux | (83) |
|  | | MI | Human ESC-CVPCs | UC | *In vivo:*  20μg | *In vivo:*  LV ejection fraction↑  LV systolic dimensions↓  Scar area↓ | Promoting angiogenesis and inhibiting cardiomyocyte death | (84) |
|  | | Cardiac hypertrophy | Human CDCs | Centrifugation filter | *In vivo:*  350μg | *In vivo:*  Cardiomyocyte cross-sectional area↓  Interstitial cardiac fibrosis↓  LV posterior wall diameter↓  LV internal diastolic diameter↑ | Delivering Y RNA fragments to induce IL-10 (a vital anti-inflammatory cytokine) expression | (87) |
|  | | DCM | Human CDCs | UC | *In vivo:*  1.0 × 10^4^ | *In vivo:*  LV fibrosis↓ | Shedding proangiogenic and cardioprotective miR-146a-5p to suppress inflammatory response | (89) |
|  | | Myocardial ischemic injury | Human RACs | UC | *In vivo:*  5×10^5^ cells equivalents after IRI 30min, day 1, day 3  *In vitro:*  2.5×10^5^ and 5×10^5^ | *In vivo:*  Mitral regurgitation severity grade↓  Interstitial fibrosis↓  Anti-fibrosis miRNA↑  *In vitro:*  Cell division cycle↑ | Delivering pro-angiogenesis, cardiomyogenesis and anti-inflammatory miRNAs | (91) |
| **Lung** | | Lipopolysaccharide | Mice BMSC | Centrifugation filter | *In vivo:*  70μg  *In vitro:* | *In vivo:*  Histopathological score↓  *In vitro:*  TGF-β1, α-SMA, COL I, COL III↓  E-cadherin↑ | Transmitting miR-23a-3p and miR-182-5p to inhibit NF-κB/Hedgehog pathway | (97) |
|  | | Experimental silicosis | Mice ADMSC | UC | *In vivo:*  50μL  *In vitro:*  3 × 10^8^ | *In vivo:*  Collagen deposition↓  Interstitial fibrosis↓ TGF-β↓  *In vitro:*  TGF-β↓ | Delivery miR-146b to suppress NF-κB/TLR pathway | (98) |
|  | | BLM | hucMSC | UC | *In vivo:*  20μg on day 7 and day 21  *In vitro:*  10ng/mL | *In vivo:*  Collagen deposition↓  α-SMA, FN, Tgfbr2 and Tgfb2↓  *In vitro:* α-SMA, Tgfbr2, Tgfb2↓ | Delivering miR-21-5p/miR-23-3p to suppress the TGF-β pathway | (99) |
| BLM | | BLM | Human BMSC | UC | *In vivo:*  100μg  *In vitro:*  10μg | *In vivo:*  α-SMA, COL I↓  *In vitro:*  α-SMA, COL I↓  G0/G1%, apoptosis rate↑ | Delivery miR-186 to downregulate of SOX4 | (100) |
|  | | BLM | Human BMSC | Iodixanol gradient ultracentrifugation | *In vivo:*  200 μL; dose, 5 × 10^6^ MSC equivalents; ~8.6 × 10^8^ particles | *In vivo:*  Collagen deposition↓  Apoptosis↓  Ccl2, Arg1, Il-6, Tgfb↓ | Reducing monocytes infiltration | (101) |
|  | | BLM | Human BECs | UC | *In vivo:*  2.0×10^9^/body on day 7 and 14  *In vitro:*  10μg/mL | *In vivo:*  Collagen deposition↓  *In vitro:*  α-SMA, COL I↓ | Inhibiting the crosstalk between TGF-β and Wnt pathways | (103) |
|  | | BLM/experimental silicosis | Human LSCs | Ultrafiltration | *In vivo:*  10×10^9^/body weight | *In vivo:*  Hemorrhagic necrosis↓  Apoptosis↓  Alveolar epithelial structures↑  lung function↑  Collagen deposition↓  α-SMA↓  Smad3, MMP-2↑ | Shedding miRNAs (miR-30a, miR-99 and let-7) | (105) |
| **Liver** | | CCl_4_ | Human amnion MSC | UC | *In vivo*:  20 μg/kg  *In vitro:*  0.5 μg/ml | *In vivo*:  Fibrosis area↓  α-SMA↓  *In vitro:*  α-Sma, Timp-1↓  Mmp-2↑ | Suppressing Kupffer cells and hepatic stellate cells activation | (108) |
|  | | CCl_4_ | Mice ADMSC | UC | *In vivo*:  40 μg, twice a week for 8 weeks | *In vivo*:  AST, ALT, TB↓  Collagen volume fraction↓  α-SMA, COL I, COL III, FN↓  *In vitro:*  α-SMA, COL I, COL III, Vimentin↓ | Transferring miR-150-5p to inhibit CXCL1 signaling | (110) |
|  | | cGVHD | Human BMSC | UC | *In vivo*:  100 μg once per week for 6 weeks | *In vivo*:  Fibrosis area↓ | Inducing IL-10^+^ regulatory cells and inhibiting IL-17^+^ pathogenic T cells | (113) |
| c | | CCl_4_ | Human iPSC | UC | *In vivo*:  1.5 × 10^6^ twice a week for 2 weeks | *In vivo:*  α-SMA, COL 1α1, TIMP-1↓  Fibrosis area↓ | Shuttling functional miRNA (*e.g.*, miR-92a-3p) | (114) |
|  | | bile duct ligation |  |  | *In vivo*:  1.5 × 10^6^ once daily for 6 days | *In vivo*:  α-SMA↓  Fibrosis area↓ |  |  |
|  | NASH | | Human LSCs | UC | *In vivo*:  5 × 10^9^; 2.5 × 10^9^; 2.5 × 10^8^ twice per week for 2 weeks | *In vivo*:  ALT↓  Peri-venous and peri-portal area↑  Fibrosis area↓  Col 1α1, α-Sma, Tgf-β1↓ | Delivering miR-29a, miR-30a and let-7 to targeted inhibiting collagen I and Snail | (116) |
|  | CCl_4_ | | primary T-MSC | UC | *In vitro*:  human pHSCs: 1, 10, and 100 μg/mL  murine pHSCs: 100 μg/mL  *In vivo*:  150 μg/mouse, once a week | *In vivo:* AST, ALT↓  α-SMA, TGF-β, Vimentin, COL 1α1, Ctgf↓  *In vitro:*  α-SMA, TGF-β, Vimentin, Ctgf↓ | Delivery of miR-486-5p to inactivate Hedgehog signaling | (117) |
|  | TGF-β1 | | NK-92MI cells | UC | *In vitro:*  10 μg/mL | *In vitro:*  α-SMA, COL 1α1↓ | Transferring miR-223 to inhibit ATG7 pathway | (120) |
|  | CCl_4_ | | mouse or human hepatocytes | Iodixanol gradient ultracentrifugation | *In vivo*:  0–80 μg/25 g three times/week over the last 2 weeks  *In vitro:*  8–16 μg/ml or 2–4 × 10^8^ particles/ml | *In vivo*:  α-SMA, COL 1α1, CTGF↓  *In vitro:*  Ctgf, Col 1α1↓ | Regulation of genes associated with ECM | (121) |
|  | HFD/CCl_4_ | | *Akkermansia muciniphila* | UC | *In vivo:*  50 μg protein/200 μl daily for 4 weeks  *In vitro:*  1, 10, 50 μg/ml | *In vivo:*  AST, ALT↓  TGF-β, α-SMA, PDGF, TIMP1, COL 1α1↓  *In vitro:*  TGF-β, α-SMA, TIMP1, COL 1α1↓ | Normalizing the gut microbiota composition disorders | (122) |
| **Kidney** | AAN | | Human BMSC | UC | *In vivo*:  1 × 10^10^ EVs/ml/mouse once a week for 4 weeks  *In vitro*:  75,000 EVs/cell | *In vivo*:  CREA, BUN↓  Interstitial fibrosis↓  Tubular necrosis↓  α-SMA, Col1a1, Tgfb1, Ltbp1↓  *In vitro*:  α-Sma, Col 1a1, Tgfb1↓ | Delivery miR-21to regulate inflammation, apoptosis and fibrosis | (126) |
|  | UUO | | hucMSC | UC | *In vivo*:  200 μg/rat  *In vitro*:  160 μg/ml | *In vivo*:  α-SMA, COL I, FAP, TGF-β1↓ | Promoting YAP degradation | (128) |
|  | DN | | Rat BMSC | UC | *In vivo*:  2 injections of 100 μg/kg/dose | *In vivo*:  Collagen fibers↓  TGF-β, FN↓ | Inducing mTOR-mediated autophagy | (130) |
|  | MetS-RAS | | Swine ADMSC | UC | *In vivo*:  1 × 10^10^ | *In vivo*:  CREA↓  Tubulointerstitial fibrosis↓  Glomerular score↓ | IL-10 dependent immunoregulation | (131) |
|  | MetS-RAS | | Swine ADMSC | UC | *In vivo*:  1 × 10^10^ | *In vivo*:  Kim-1, COL I↓  Tubular injury score↓  Fibrosis area↓ | Inducing regulatory T cells | (132) |
|  | RVD | | Swine ADMSC | UC | *In vivo*:  1 × 10^11^, approximately 100 µg of protein | *In vivo*:  CREA↓  Tubular injury score↓  Tubulointerstitial fibrosis↓  Glomerulosclerosis↓  GFR↑  Peritubular capillary density↑ | Delivering vasculo-protective genes | (133) |
|  | Ischemic AKI | | mice ESC | UC | *In vivo*:  100 μg | *In vivo*:  CREA, BUN↓  α-SMA, Kim-1↓ | Promoting TECs proliferation and angiogenesis | (134) |
|  | AAN | | Human LSCs | UC | *In vivo:*  1 × 10^10^ EVs/mL/mouse per week for 4 weeks  *In vitro*:  50,000 EVs/cell | *In vivo:*  NGAL↓  Interstitial fibrosis↓  *In vitro*:  α-Sma, Col 1α1↓ | miR-29b-mediated inhibition of Wnt/β-catenin pathway | (135) |
|  | Ischemic AKI | | Rat primary renal tubular cells | UC | *In vivo*:  100 μg at 24 and 48 hours | *In vivo:*  CREA↓  COL I, COL II, COL IV, COL V, α-SMA, FN↓ | Delivering ribosomal transcripts | (136) |
|  | CIN | | Rat plasma | Exosome Isolation Reagent | *In vitro:*  coculture | *In vivo:*  CREA, BUN↓  Kim-1↓  Fibrosis area↓ | exosomal miR-1-3p to target ATG13 and activate the AKT pathway | (137) |
| **Others** | IUA | | Rabbit BMSC | Total Exosome Isolation Reagent | *In vivo:*  50μg  *In vitro:*  25μg/mL,50μg/mL, and 100μg/mL | *In vivo:*  Glands of the endometrial uterus↑  CK19, Vimentin↑  Fibrosis area↓  TGF-β1, TGF-βR1, and Smad2↓  *In vitro:*  CK19, E-cadherin↑  FSP1, Vimentin, TGF-β1, TGF-βR1, Smad2↓ | Reversing EMT via TGF-β1/Smad2 pathway | (138) |
|  | Subretinal fibrosis | | hucMSC | UC | *In vivo:*  2μL on days 7, 21, and 35 | *In vivo:*  Choroidal neovascularization↓  Fibrosis area↓  *In vitro:*  α-SMA, occludin, Vimentin, N-cadherin, zonula occludens-1↓ | miR-27-3p to suppressed the activation of EMT by targeting HOXC6 | (140) |
|  | IDD | | Rat BMSC | UC |  | *In vitro:*  ECM degeneration↓  Fibrosis deposition↓  COL I↓ | Delivering miR-532-5p to inhibit the pro-apoptotic RASSF5 pathway | (141) |
|  | Urethral stricture | | hucMSC | UC | *In vivo:*  30μg  *In vitro:*  10μg/mL | *In vivo:*  α-SMA↓  collagen fibers↓  *In vitro:*  α-SMA, COL I, COL III↓  IL-6, IL-1β↓ | Shedding miR-146a to inhibit pro-inflammatory pathways | (142) |

Abbreviation: ADMSC: adipose-derived MSC, UC: ultracentrifugation, MI: myocardial infarction, LV: left ventricle, α-SMA: α-smooth muscle actin, COL: collagen, eNOS: endothelial nitric oxide synthase, iPSC-CM: iPSC-derived cardiac cells., MMP: matrix metalloproteinase, ESC-CVPCs: embryonic stem cell derived cardiovascular progenitor cells, CDCs: cardiosphere-derived cells, DCM: dilated cardiomyopathy, RACs: regeneration-associated cells, BMSC: bone marrow derived MSC, TLR: Toll like receptor, BLM: bleomycin, FN: fibronectin, Tgfbr2: tgf-β receptor 2, SOX4: SRY- related HMG box transcription factor 4, Ccl2: monocyte chemoattractant protein-1, Arg1: Arginase 1, BECs: bronchial epithelial cells, LSCs: lung spheroid cells, CCL4: carbon tetrachloride, TIMP: tissue inhibitors of matrix metalloproteinases, AST: aspartate aminotransferase, ALT: alanine aminotransferase, TB: total bilirubin, cGVHD: chronic graft-versus-host disease, CXCL1: CXC chemokine-ligand-1, ATG: core autophagy-related genes, HFD: high-fat diet, AAN: aristolochic acid nephropathy, CREA: creatinine, BUN: blood urea nitrogen, UUO: unilateral ureteral obstruction, hucMSC: human umbilical cord MSC, FAP: fibrosis-related proteins, YAP: Yes-associated protein, DN: diabetic nephropathy, mTOR: mammalian target of rapamycin, MetS-RAS: metabolic syndrome- renal artery stenosis, Kim-1: kidney injury marker-1, RVD: Renovascular disease, GFR: glomerular filtration rate, AKI: acute kidney injury, ESC: embryonic stem cell, NGAL: neutrophil gelatinase-associated lipocalin, CIN: contrast-induced nephropathy, AKT: protein kinase B, IUA: intrauterine adhesion, FSP1: fibroblast-specific protein-1, HOXC6: homeobox protein Hox-C6, IDD: intervertebral disc degeneration, RASSF5: Ras association domain family member 5.
